# Case of Meningeal Apoplexy, with Some Remarkable Phenomena, and the Appearances on Dissection

**Published:** 1824-05

**Authors:** James Johnson

**Affiliations:** Editor of the Medico-Chirurgical Review, &c. &c.


ART. II.—*Case of Meningeal Apoplexy, with some remarkable Phenomena, and the Appearances on Dissection.* By JAMES JOHNSON, M.D. Editor of the Medico-Chirurgical Review, &c. &c.

LADY D—, aged sixty-two years, of full habit, and rather corpulent, residing in New-street, Spring Gardens, had been out in her carriage on Tuesday, the 10th December, 1822, in very good health and spirits. At four P.M. she returned, and went into the back drawing-room, to finish a letter. At six o'clock her husband happened to look into the room, and saw her rolling on the floor in a state of insensibility. Sir Astley Cooper and Dr. Johnson were called to her in a few minutes, and Dr. Warren and Mr. Freeman soon after arrived. When placed on a sofa, Lady D— kept constantly throwing about her arms and legs, rolling her head, and sighing. She was insensible when spoken to; her pulse was very unequal, and weak; her skin cool; her face pale; the pupils moderately sized, and obedient to the stimulus of light. Sir Astley Cooper opened a brachial vein, but not more than an ounce of blood flowed. The pulse now fell considerably; the respiration became laborious; the muscular agitation subsided, and she appeared to be dying. On pouring down the throat some hartshorn and water, a slight effort to vomit ensued, and the pulse acquired some force. Ten grains of the sulphas zinci were administered, and mustard cataplasms applied to the feet. In a few minutes more, a similar dose of the sulphas zinci were administered, but no effect was produced on the stomach. Another vein was opened by Mr. Freeman, and two ounces of blood procured. The temporal artery was next divided, but, not bleeding to any extent, the aural branch was opened, and thirty-six ounces of blood were abstracted. The pulse now began to flag, and the artery was secured. On speaking loudly to the patient, she now evinced some degree of sensibility, and answered a few questions indistinctly, relapsing immediately into a state exactly resembling common sleep.

She was now removed into bed; ten grains of calomel were administered with some difficulty, and an assafœtida enema thrown up. She was ordered to have five grains of calomel and a black draught every three hours, till the bowels were freely moved. No evacuation took place through the night, and at nine o'clock next morning, the pulse having got up, and the coma continuing, a vein was opened, and sixteen or eighteen ounces of blood abstracted. The pulse now fell, and became irregular ; the respiration laborious ; and some stertor appeared for the first time. These symptoms, however, disappeared in a considerable degree; the bowels became freely opened ; much sensibility returned, so that she answered several questions; and the pulse became regular, forcible, and about eighty in number. In the course of this day, (the second of the apoplexy,) the sinapisms occasioned so much distress, that it required several attendants to confine the patient in her struggles to get out of bed. In these attempts she sometimes sat almost upright in bed, moaning, and uttering broken accents of complaint. The sinapisms were therefore removed ; soon after which the patient became tranquil, and fell into a soporose state. During the whole of this day, the pupils exhibited no particular deviation from a natural condition, being more or less obedient to the stimulus of light. No paralysis or drawing of the face had yet appeared, the patient being still able to swallow liquids.

In the evening of this day (Wednesday), the coma having become more marked, and the pulse having risen above 100 in the minute, with considerable strength, cupping-glasses were applied to the temples at half-past nine o'clock, and about sixteen ounces of blood were abstracted ; when the pulse began to flag.

At this time so much sensibility returned, that the patient answered several questions, knew some of her relations, and called them by their names. Very soon after ten o'clock, however, the coma recurred, the breathing became laborious and stertorous, and the pulse irregular. The patient continued to move the head, and also the extremities, till three o'clock in the morning of Thursday, (thirty-three hours from the beginning of the attack,) when all voluntary power, except deglutition, *appeared* to be lost, and she lay in a state of coma, with stertorous breathing, till half-past eight o'clock on Thursday evening, or about fifty-one hours from the commencement of the apoplexy, and eighteen hours after the apparent general paralysis of the voluntary muscles took place. During these eighteen hours she swallowed several spoonfuls of drink, and some medicines.

*Dissection.*—Sir Astley Cooper and Mr. Freeman opened the head on Friday evening, twenty-four hours after death; and Dr. Warren and Dr. Johnson were also present.

The dura mater presented no unusual appearance. There was some opacity, as well as thickening, of the tunica arachnoidea. Between this membrane and the pia mater there was a moderate effusion of serum, but quite sufficient to raise it from the subjacent tissue, and render its opacity perceptible. The pia mater, wherever it extended, (over the whole of the hemispheres, between them, among the convolutions, and over the base of the brain, medulla oblongata, and as far as could be seen of the spinal marrow,) was infiltrated with black blood, very much resembling in appearance the tunica conjunctiva of the eye, when highly *bloodshot.* This state was very different from that of mere congestion of the vessels. No blood-vessels, whether veins or arteries, were distinguishable, but the texture of the pia mater seemed completely impregnated or infiltrated with blood, so as not only to change its natural appearance, but to render its vessels undistinguishable by the eye. Besides this *bloodshot* condition of the pia mater, there was a considerable quantity of blood distinctly extravasated in globular coagula, dispersed over various parts of the inner surface of the membrane, but more particularly between the hemispheres, among the convolutions, and at the base of the brain. These coagula were of various sizes, from that of a pin's head to the size of a small pea, but not larger. Wherever the pia mater was stripped off the brain, these graniform extravasations rose with it, leaving the surface of the brain clean and of its natural colour. The brain itself was softer than natural, and exhibited no marks of increased vascularity. No red points were observable when it was sliced. In the two lateral ventricles there was about an ounce of very red serum, and a small quantity in each of the other two ventricles, and at the base of the brain; the whole serous effusion amounting, perhaps, to two ounces. The pia mater covering the cerebellum was in exactly the same state as that covering the different parts of the cerebrum. The lining of the ventricles had a blush of red, but nothing like the bloodshot appearance of the pia mater.

In the basilary artery, and in the circulus Willisii, there were points of calcareous deposition, but no vessel of any magnitude was ruptured within the head.

It may be proper to state that, for some months before the fatal event, Lady D— had been subject to head-aches, and for years was affected with constipated bowels. For some weeks, or perhaps months, she had also been under considerable mental anxiety, on account of some near relatives. These circumstances may fairly be considered as predisposing to the apoplectic attack of which she died.

This dissection has afforded one of the most exquisitely-marked specimens, perhaps, on record of what may be properly termed *meningeal apoplexy*, as distinguished from cerebral or cerebellic. In this case there is no rupture of any particular vessel,—no accumulation of blood in any particular part,—no laceration or excavation of any portion of brain,—no partial, though considerable general, pressure. The symptoms were in strict accordance with these pathological phenomena. The uniform pressure over the whole encephalon, produced by this bloodshot state of the pia mater and diffused extravasation, was sufficient to abolish, or at least to suspend, the intellectual operations, without depriving the muscular system, whether voluntary or automatic, of its moving powers.

It was seen that, when the effects of this general pressure on the brain were somewhat mitigated by the abstraction of thirty-six ounces of blood, perception and intellectuality were so far restored, that the patient answered questions with some degree of distinctness; a circumstance that was still more evident on the evening of the second day, when sixteen ounces of blood were abstracted from the temples.

It has been stated by a recent writer on Apoplexy (Mr. Serres), that the distinction between meningeal apoplexy and cerebral may be drawn in the living body, by finding the absence of paralysis, and the presence of sensibility, to proper stimuli in the former (meningeal); and some degree of paralysis in one or other side, or drawing of the mouth, in the latter, or cerebral. If it be objected, that in this case there was general paralysis during the last eighteen hours of the patient's life, it may be fairly urged, on the other hand, that there are great doubts respecting the actual existence of this general paralysis. The degree of stupor was probably so great, as to prevent the sinapism to the precordia, or any uneasiness in position, from exciting the voluntary muscles to motion. That the paralysis was not general during this period, is proved by the power of deglutition (which is a voluntary power,) remaining till almost the last moment. In some cases of heavy sleep, or drunkenness, where there is no paralysis, the same degree of stimulation used in the last eighteen hours of this apoplectic state, would produce no exertion of the voluntary muscles. We have not, therefore, any proof of paralysis, strictly so called, (and to be distinguished from the gradual abolition of all vital movements, which precedes death,) in this remarkable case; and so far it tends to prove the justness of the criterion laid down by Mr. Serres, between meningeal and cerebral apoplexy. In one hundred cases of fatal apoplexy at La Pitié, which were carefully watched and afterwards examined, twenty-one were uncomplicated with any paralysis; and in none of these was there any laceration of the brain, or local extravasation of blood. Sixteen of these presented *serous* effusion, either in the ventricles or convolutions of the brain; one with sero-sanguineous effusion in the left lateral ventricle; two with a similar effusion between the arachnoid and pia mater; and two without any effusion at all, but only turgescence or congestion of the vessels. In every one of the seventy-nine cases, where there was paralysis of one or more members, or drawing of the mouth to one side, there was found local extravasation of blood in some part of the brain.*

The case which is here presented affords a very exquisite example of what is termed a local determination to a single tissue of an organ, leaving all the other structures, as it were, untouched. It has been called a *coup de sang* by the French pathologists, which corresponds with *bloodshot,* the expression here applied, and which conveys a more accurate idea of the appearances than any other term. It is worthy of notice that the tunica arachnoidea, which is the most delicate structure of the meninges, exhibited traces of inflammatory action,—viz. the opacity, and perhaps the serous effusion between it and the pia mater. In explanation of this phenomenon, it must be remembered that, notwithstanding the evacuations, there was considerable excitement manifested in the vascular system during a great portion of Wednesday, the second day of the attack, the pulse having got above 100 in the minute, attended with sharpness, while the pulsations in the carotids were very strong and hard. There is fair reason to suppose that, during this re-action and the great determination of blood to the head, the arachnoid appearances were produced. It was the opinion of one of the medical attendants that the apparent general paralysis, or abolition of voluntary movements, in the morning of

* See Dr. Cooke's valuable work on Palsy, page 70 to 84.

Thursday, or eighteen hours before death, corresponded with the effusion into the ventricles. The narrator of this case hesitates to adopt this opinion; and is rather inclined to attribute the general quiescence of the voluntary muscles during the period above mentioned, to the general progress of the infiltration of the pia mater, and the effects of universal pressure on the brain from the meninges without, and the ventricular effusions within. This and other points, however, may possibly claim the attention of some of your readers, and form, perhaps, a topic of illustration, for those who have directed their researches to this very interesting pathological subject.

